# The Older Driver with Cognitive Impairment: Perceptions of Driving Ability and Results of a Behind the Wheel Test

**DOI:** 10.3390/geriatrics1010006

**Published:** 2016-02-04

**Authors:** Laura Hemmy, Susan Rottunda, Geri Adler

**Affiliations:** 1 Geriatric Research, Education, and Clinical Center (GRECC), VA Health Care System, One Veterans Drive, Minneapolis, MN 55417, USA; Susan.Rottunda@va.gov; 2 Department of Psychiatry, University of Minnesota, Minneapolis, MN 55414, USA; 3 Veterans Affairs South Central Mental Illness Research, Education, and Clinical Center (MIRECC), 2002 Holcombe Blvd., Houston, TX 77030, USA; Geri.Adler@va.gov; 4 Houston VA Health Services Research & Development Center for Innovations in Quality, Effectiveness and Safety, Michael E. DeBakey VA Medical Center, Houston, TX 77030, USA; 5 Menninger Department of Psychiatry and Behavioral Sciences, Baylor College of Medicine, Houston, TX 77030, USA

**Keywords:** older drivers, caregivers, assessment, cognitive impairment

## Abstract

Older adult drivers with cognitive impairment pose a potential safety risk to themselves and others. Providers are often uncertain about when to request a formal evaluation of driving ability, leaving subjective reports of concerns by the patient or family as common initiators of objective driving evaluation referral. This observational study evaluated the correspondence of patient and caregiver report of driving concerns relative to objective behind-the-wheel (BTW) testing. Data were analyzed from occupational therapy driving evaluations of older adult U.S. Veterans referred from cognitive disorder specialty clinics between 2005 and 2015 (*n* = 151). Driving ability was evaluated with a pre-testing interview of the patient and a knowledgeable caregiver, followed by objective BTW testing. Patients referred had a mean age of 77.6 (SD = 8.1) years, were 97% male, and 98% white. Results demonstrated that most patients are evaluated for driving concerns far too late, with only 3% of the sample being evaluated as independent to drive without restrictions, and 38% recommended to retire from driving. Although both patients and caregivers denied specific driving concerns (obey signs and lights) relative to objective testing, caregiver concerns were greater than their respective patient’s concerns (*p* < 0.001) and were associated with road test outcome (*p* = 0.001).

## 1. Introduction

Older adult drivers with cognitive impairment pose a potential safety risk to themselves and others. Consequently, concerns about older drivers with cognitive impairment continue to increase, and the need to identify impaired drivers cannot be underestimated. Progressive neurodegenerative disease leading to dementia, such as Alzheimer’s disease (AD), is the most common cause of functionally disabling cognitive impairment in older adults [1]. Thus, for the majority older adult drivers, the onset of cognitive impairment is an indication of future decline in cognitive and functional abilities, including the ability to drive. Unlike many traditional occupational therapy or rehabilitation services, the goals of driving assessment and intervention in a progressively declining patient population are to predict and facilitate when and how to help patients limit and eventually retire from driving. 

Inevitably, most older adults with cognitive impairment will have to stop driving. However, research findings regarding the safety of drivers in the early stages of cognitive impairment from neurodegenerative disease are mixed. In a 2000 evidence-based review, researchers concluded that drivers with mild dementia (Clinical Dementia Rating (CDR) score = 1.0) were not safe to drive [2]. However, in a 2005 study of drivers with early AD, it was found that 85% of subjects with very mild AD (CDR = 0.5) and 76% of subjects with mild AD (CDR = 1.0) were able to pass a behind the wheel (BTW) test [3]. 

Despite the knowledge of at least some cognitive impairment, several studies have found that approximately one third of patients with dementia continue to drive [4,5,6]. Patients, caregivers, and health care providers are often uncertain about when to request a formal evaluation of driving ability, even though subjective reports of driving concerns by the patient or family may be the primary initiating factors for objective driving evaluation referral. For example, one survey found that caregiver driving concerns were a particularly strong contributor to physicians’ decision to report the patient to licensing authorities [7].

Countries rely on regulatory systems to ensure older drivers are fit to drive, with the degree of oversight regarding age and/or medical conditions varying widely. For example, Britain requires drivers aged 70 and older to self-certify their own fitness to drive [8], while Finland requires formal medical examinations of all drivers starting at age 70 [9]. In the United States (U.S.), regulations are made locally by states, with some requiring in-person renewal after a certain age. Presently, only a few states have mandatory medical reporting for drivers with decreased capacity (California, Delaware, Nevada, New Jersey, Oregon, and Pennsylvania) [10]. Dugan and colleagues [11] found in their review of U.S. policies to improve older driver safety that in-person renewal and visual testing at renewal lead to a reduction in fatal crashes, whereas restricted licenses and knowledge tests did not significantly reduce crash risk.

Drivers with cognitive impairment may lack insight into their decreased abilities, and thus be less willing to accept provider recommendations to restrict or retire from driving [12,13]. Caregivers may also not recognize that the patient is unsafe to drive. Alternatively, they may recognize changes in the patient’s cognition or driving performance, but do not feel comfortable discussing it with their loved one [14]. In a study comparing the ability of providers and caregivers to predict BTW driving ability of patients with AD, provider ratings were significantly correlated with the patient’s BTW road test performance, but caregiver ratings were not [15]. Poor insight in regard to how dementia impairs driving ability is further reflected by the results of a 1999 survey of drivers with a dementia diagnosis and their caregivers. Sixty-five percent of dementia patients and 43% of caregivers surveyed did not believe a diagnosis of dementia would result in a need to stop driving [16].

These findings suggest there may be considerable limitations of both patient and caregiver report of patient driving ability in the context of dementia. This is despite the fact that such report may be the initiator of safety evaluation and access to services. Additional data are needed to help providers evaluate the validity of patient and caregiver report in regard to driving ability. This observational study evaluated the correspondence of patient and caregiver report of driving concerns relative to each other, and to objective BTW testing. Data were analyzed from occupational therapy driving evaluations of older adult U.S. Veterans referred between 2005 and 2015 from cognitive disorder specialty clinics primarily serving patients with AD and other common neurodegenerative dementias.

## 2. Methods

### 2.1. Participants

Data were obtained from the records of older adult Veterans referred for occupational therapy (OT) driving evaluation by cognitive specialty clinics (Geriatric Research Education and Clinical Center Memory Clinic and Dementia Care Coordination Clinic) at a Midwestern VA Medical Center between 2005 and 2015. Only those Veterans having completed a pre-driving test interview and also initially assessed by the OT evaluator as safe to complete the BTW portion of the evaluation were included. Caregiver interview data were also obtained from these same evaluations. All records were retrieved with review and approval by the institutional committee for the protection of human subjects.

The resulting participants consisted of 151 Veteran patients (and their caregivers) with a mean age of 77.6 (SD = 8.1) years, 97% male (147/151), and 98% (148/151) white. Of those meeting inclusion criteria (referred by a cognitive specialty clinic, and having completed both the pre-test interview and a BTW test), 108 had been evaluated with the MMSE in the course of their care in the referring specialty clinic. The mean MMSE score for this subsample was 22.6 (SD = 4.9).

All participants were documented as possessing a currently valid driver’s license or permit. Of those OT evaluators reporting patient license restriction data, 71% (93/131) of patients had licenses with some pre-existing driving restrictions in place. Pre-evaluation license restriction data are presented in Table 1. Of those caregivers reporting their own driving status, 82% (102/125) reported they are also current drivers and not dependent on the patient to drive.

**Table 1:** Current license restrictions in order of frequency.

Restriction	N	Yes	%
Corrective lenses required	131	91	69.5
Mirrors or other adaptive equipment required	118	3	2.5
Daytime driving only	118	2	1.7
Speed zone restriction	118	1	0.8
Radius or local area restrictions	118	1	0.8

### 2.2. Procedures

The authors reviewed the records of patients who completed an OT-administered comprehensive driving evaluation that included a pre-testing interview, as well as an objective BTW test. The interview included questions for the patient and caregiver about the patient’s current driving habits, any self-imposed driving restrictions (see Table 2), and any concerns either had about the patient’s driving (both global and specific ability questions; see Table 3). Not all OT evaluators included all interview questions, and not all patients or caregivers chose to respond to every question. 

Objective BTW testing was administered by OT Driving Rehabilitation Specialists (OT/DRS) and completed in stages to ensure the safety of both patient and evaluator. After providing an orientation to the vehicle, patients were asked to start driving in a parking lot. If able to demonstrate safe driving behavior, s/he was allowed to progress to more difficult driving environments including residential streets, highways, and freeway driving. The BTW assessment was discontinued if the OT/DRS determined that it was unsafe, and the patient was considered to be unable to complete the more challenging elements of the assessment. BTW assessment elements are standardized across evaluators and include the following: (1) use of controls, turn signals, and mirrors; (2) blind spot checks; (3) turn accuracy; (4) identify, and obey traffic signs; (5) intersection safety; (6) uncontrolled left turn; (7) tracking; (8) lane changes; (9) backing skills; (10) speed limit check; (11) merging; (12) enter/exiting of freeway; (13) following distance; and (14) parking. Upon completion of the examination, the OT/DRS made one of three outcome recommendations: (1) unrestricted driving; (2) continued driving with added restrictions; or (3) retire from driving. Examples of driving restrictions included daytime driving only, area or destination restrictions (e.g., local or grocery store), and no freeway driving.

### 2.3. Data Analysis

Descriptive statistics are reported about the characteristics of the sample, pre-test interview responses, and outcome of BTW behavioral test performance. When descriptive percentages of interview responses are reported, all respondent data available (patients and/or caregivers) are reported, regardless of whether their paired dyad (e.g., patient-caregiver) responded to the same interview items. These values represent mean interview responses by class of individual reporting (patient *vs.* caregiver), but not by the dyad unit. In the case of direct statistical comparisons of patients’ reporting relative to caregivers’ reporting, paired samples *t-*tests are presented, representing a comparison within the dyad unit (*i.e.*, did the patient and caregiver agree with respect to the same patient’s behavior?)

Differences in the mean percentage of reported driving concerns (patients and caregivers, individually) across patients with differing driving evaluation outcomes are evaluated with univariate ANOVAs. Possible driving evaluation outcomes were collapsed from three (driving without restrictions *vs.* driving with restrictions *vs.* retire from driving) to two (continued driving with or without restrictions *vs.* retire from driving) for these analyses, due to the infrequency in which patients were recommended to continue driving without restrictions.

The interview item about obeying traffic signs and lights is the only item with a specific behavioral correlate included as part of the BTW test. Relationships between responses to this interview item (patients and caregivers, individually) and signs/lights road test performance were evaluated with chi-square tests. Lastly, the relationship between patients’ ability to obey signs and lights during the BTW test and final driving outcome recommendation (continued driving with or without restrictions *vs.* retire from driving) were also evaluated with chi-square tests.

Analyses were conducted using 2-sided tests, with *p* ≤ 0.05 as the criterion for statistical significance. Cases with missing values were omitted from each individual analysis in which that value was included, but maintained in any others for which data were available. All statistical analyses were conducted using SPSS [17].

## 3. Results

### 3.1. Pre-BTW Interview

#### 3.1.1. Patient-Reported Driving Restrictions

Despite the fact that few patients had specific restrictions on their licenses, other than required vision correction, a fair number of patients endorsed already having begun to limit or self-restrict their driving in some way. Table 2 presents the patients’ pre-evaluation driving restrictions as self-reported in the interview. Note, there was no independent corroboration of whether the patients’ actual driving behaviors matched their self-reported driving habits.

**Table 2:** Patient-reported driving restrictions in order of frequency.

Driving Restriction Items	N	No	%
Patient drives all distances	132	47	35.6
Patient drives both day and night	141	42	29.8
Patient drives in all weather conditions	127	31	24.4
Patient drives on freeways	138	24	17.4
Patient drives on highways	139	11	7.9
Patient drives in commercial areas	137	4	2.9
Patient drives in residential areas	141	1	0.7

#### 3.1.2. Patient- and Caregiver-Reported Driving Concerns

Ninety-eight percent (148/151) of patients and 89% (134/151) of caregivers responded to at least one pre-BTW interview question about the patient’s driving. The mean number of interview questions describing concerns about patients’ driving that each responded to were 5.3 (SD = 2.1) and 5.1 (SD = 2.7), respectively, for patients and caregivers. These interview items, along with patient and caregiver responses, are presented Table 3. The mean percentage of items endorsed in the “yes” direction (indicating driving concerns) relative to those items answered, were 11.1% (SD = 18.1) for patients and 38.6% (SD = 35.4) for caregivers. When compared as a paired sample (i.e., matching patients with their respective caregivers), caregivers were reporting a significantly greater proportion of driving concerns about the patient’s driving behavior (*t*(130) = 8.52, *p* < 0.001).

**Table 3:** Pre-BTW interview responses for patients and caregivers.

Interview Items - Yes/No Response Format	Patients			Caregivers		
	N	Yes	%	N	Yes	%
Reports concerns about the patient’s driving	144	14	9.7	134	97	72.4
Reports accidents or moving violations in last year	144	32	22.2	93	14	15.1
Reports “near misses” in last year	137	18	13.1	90	17	18.9
Reports concerns about getting lost while driving	92	9	9.8	95	32	33.7
Reports concerns about being more distracted while driving	88	2	2.3	91	13	14.3
Reports trouble identifying or obeying traffic signs/lights	89	2	2.2	90	15	16.7
Reports the patient uses a “co-pilot” when driving	86	1	1.2	88	15	17.0
Do you feel comfortable riding when the patient is driving? *	--	--	--	94	64	68.1

Note: The patient and caregiver questions were phrased to refer to concerns about the patient’s driving; * This item was only asked of caregivers.

### 3.2. BTW Outcomes

#### 3.2.1. OT/DRS Driving Evaluation Outcome

Only five patients in the sample (3.3%) were evaluated as appropriate for future unrestricted driving as a result of the BTW test. Eighty-nine (58.9%) were evaluated as candidates to drive with some additional restrictions in place, and 57 (37.7%) were recommended to retire from driving. For those patients evaluated as appropriate for driving with added restrictions, the most common OT/DRS recommended driving restrictions are presented in Table 4.

**Table 4:** OT/DRS recommended driving restrictions in order of frequency.

OT/DRS Recommended Restriction	N	Yes	%
Daytime driving only	83	68	81.9
Area/distance restriction	86	67	77.9
Limit environmental distractions	86	52	60.5
Non-peak traffic times	80	46	57.5
No freeway driving	78	38	48.7
Speed restrictions	81	29	35.8
Use navigation assistance	75	20	26.7
Wear sunglasses while driving	77	3	3.9

#### 3.2.2. Interview Report Correspondence to BTW Testing

The percentage of interview items endorsed in the “yes” direction (indicating driving concerns) relative to those items answered, were compared across the two primary driving evaluation outcomes: recommendation to continue driving (with or without required restrictions) or recommendation to retire from driving. Patient reported percentage of driving concerns were not significantly different across driving outcomes (12.1% *vs.* 10.5%). However, caregiver reported percentage of concerns were significantly greater for those patients who were advised to retire from driving *vs.* those who were not (51.2% *vs.* 31.3%; *F*(1,132) = 10.58, *p* = 0.001).

Patients’ and caregivers’ reported driving concerns were further compared against the patient’s BTW test driving performance for the one item specifically included in both parts of the evaluation: the ability to obey traffic signs and lights. Despite the fact that only 2.2% and 16.7% of patients and caregivers (respectively) endorsed concerns about the patients’ ability to identify and obey traffic signs and lights, 71.1% (106/149) of patients evaluated failed to obey traffic signs or lights encountered during BTW testing. Neither patient nor caregiver response to the signs and lights interview item significantly corresponded to patient BTW performance. However, patient inability to independently respond to signs and/or lights during the BTW test was significantly associated with final driving recommendation (*X^2^*(2, N = 149) = 32.0, *p* < 0.001), with all but one of the recommended driving retirees making sign or light errors during the BTW test.

## 4. Discussion

Of the 151 patients receiving a BTW driving evaluation and interview, only three percent were recommended to continue driving without restriction, and 38% were recommended to stop driving completely. These BTW results, in combination with a mean MMSE of 22.6 for those in the sample, indicate that many older patients with progressive cognitive impairment are first referred for driving evaluation already beyond the point that they are safe to drive. These results are consistent with prior reports of BTW driving evaluations in older adult patients with cognitive impairment. Two studies of samples with comparable mean MMSE scores (both 25.1) reported BTW evaluation failure rates even greater than that in present sample, 56.5% [18] and 54.7% [15]. Given the high level of impairment found in the present study, it is noteworthy that only 5.8% of participants had license restrictions other than a requirement for corrective lenses.

Patients, and to some extent caregivers, lacked insight into their driving deficits. While nearly three quarters (72.4%) of caregivers reported general concern about the patient’s driving, only 9.7% of patients reported such concern. Similarly, caregivers endorsed concerns about specific patient driving behaviors on 38.6% of the total interview items, whereas patients only did so on 11.1% of the items. There were discrepancies noted in responses to interview items, not only between the type of informant (patient *vs.* caregiver), but also within informant reporting. For example, even though patients denied both general driving concerns and more specific driving deficits, 22.2% endorsed having had an accident or moving violation in the past year, and 13.1% a near miss in the past year. Caregivers also responded in a somewhat inconsistent manner with 72.4% having reported being concerned about the patient’s driving, but 68.1% said they were still comfortable riding with the patient as driver.

When compared with objective BTW driving performance (pass or fail), patient-reported percentage of driving concerns did not differ. While caregiver-reported percentage of driving concerns appeared to minimize patient deficit, they were significantly related to BTW test outcomes. When narrowed down to a specific driving behavior (*i.e.*, obeying traffic signs and lights), both patients and caregivers still substantially under-reported patient driving problems (2.2% and 16.7%, respectively, endorsed problems) relative to deficits on the BTW test (71.1%). These discrepancies within interview reporting and in comparison to BTW evaluation outcomes clearly demonstrate the limitation of patient self-report and caregiver report, and the importance of objective BTW assessment for drivers with cognitive impairment.

These results support the need for objective evaluation, and the inadequacy of waiting for patient or family member complaints before considering the possibility of impaired driving. For example, the ability to identify and obey traffic signs is a critical element of safe driving. Of those who were recommended to retire from driving, all but one failed to successfully obey traffic signs and lights. Patients had significant difficulty with this component of the BTW test, and neither patients nor caregivers reported they were aware of this deficit.

This illustrates the need for providers to address driving early in the course of cognitive decline, so that patients and their families are aware of the potential for unsafe driving. Because it is inevitable that older adults with cognitive impairment will eventually have to retire from driving, a proactive approach would allow the patient and family time to limit driving incrementally and prepare for the lifestyle adjustments associated with driving cessation. However, research has shown that a typical evaluation of an older adult at routine primary care clinic visit will not identify cognitive impairment in its early stages [19,20,21]. The under diagnosis of cognitive impairment in older adults is a major impediment to addressing driving safety concerns early.

Although the BTW test is often viewed as the criterion standard for assessing driving fitness, impaired drivers may be more readily identified in clinical practice by alternate assessment strategies. BTW tests are expensive, not usually covered by Medicare or insurance, time consuming, and may not be available or acceptable to all patients. Moreover, there are concerns of taking severely impaired drivers into uncontrolled traffic situations. Most of the conditions affecting driving safety are progressive, so a driver who performs well on the road test at one point may be unsafe months later and require reevaluation. Therefore, office-based screening or alternate technologies that predict driving performance (e.g., cognitive tests, simulators or naturalistic observation) are still needed [22,23].

Despite clear denial of driving deficits, there is some reason to suspect older adults with cognitive impairment may still harbor concerns about their driving. Although patients in the present study uniformly failed to endorse driving concerns in the evaluation interview, they did report already having begun to self-restrict their driving habits. It may be that patient failure to report driving concerns represents a combination of lack of insight and reluctance to admit to declining functioning in the health care setting. Patient reported self-restrictions were very close to those driving restrictions recommended by the OT/DRS evaluators. In both cases distance limitations and daytime-only driving were two of the top three restrictions. Older drivers may choose to limit driving exposure in this manner due to a variety of non-cognitive reasons (e.g., vision), also making it more socially acceptable for the cognitively impaired driver to do the same. Although encouraging, it is uncertain whether self- or other- imposed driving limitations will truly result in improved safety, especially since a review of U.S. policies found that restricted licenses did not significantly reduce crash risk [11].

## 5. Conclusions

Older adults with cognitive impairment are referred for objective driving evaluation far too late in the course of decline, with most patients already demonstrating impaired driving skills and lack of insight into their deficits. Even though caregivers demonstrated better insight than patients, they were still reluctant to report, or were unaware, of the extent of the patient’s deficits. These data should help guide providers in the evaluation of patient and caregiver report of driving ability. Referral for a driving screen or consultation as soon as cognitive impairment is detected may be warranted. Clearly, waiting for a patient or caregiver initiated complaint is not early enough to protect the safety of patients or the driving public. Earlier detection of cognitive impairment in older adults could facilitate greater acceptance of how these deficits are likely to compromise driving ability, allow for the initiation of discussions about driving, and promote patient involvement in planning for cessation, before the patient is substantially impaired. There may be potential for patients to be more amenable to driving limitation and planning for cessation, if involved early, and offered socially sanctioned avenues for making these behavioral changes.

Limitations of the study include the use of observational data taken from evaluations designed for clinical use and not tailored for research. The road test used, although standardized, has not been externally validated. The pre-BTW interview items administered to patients and caregivers were not specifically aligned with the BTW testing elements. Finally, the sample was comprised of mostly white males.

Strengths include a large sample of patients with known cognitive impairment at initial referral, both patient- and caregiver-report of perceived driving deficits during interview, and BTW road test performance.
